# Exopolysaccharides from *Limosilactobacillus reuteri*: their influence on in vitro activation of porcine monocyte-derived dendritic cells - brief report

**DOI:** 10.1007/s11259-024-10445-6

**Published:** 2024-07-04

**Authors:** Zuzana Kiššová, Petra Schusterová, Dagmar Mudroňová, Jaroslav Novotný, Ľudmila Tkáčiková

**Affiliations:** 1 Department of Morphological Disciplines, University of Veterinary Medicine and Pharmacy in Kosice, Komenského 73, 041 81 Košice, Slovakia; 2 Department of Microbiology and Immunology, University of Veterinary Medicine and Pharmacy in Kosice, Komenského 73, 041 81 Košice, Slovakia; 3 Clinic of Swine, University Veterinary Hospital, University of Veterinary Medicine and Pharmacy in Kosice, Komenského 73, 041 81 Košice, Slovakia

**Keywords:** Dendritic cells, Immune response, Cytokines, *Limosilactobacillus reuteri*, Exopolysaccharides

## Abstract

The aim of this study was to evaluate the immunomodulatory potential of two α-D-glucans from *Limosilactobacillus reuteri* L26 Biocenol™ (EPS-L26) and *L. reuteri* DSM17938 (EPS-DSM17938), with respect to their influence on in vitro activation of porcine dendritic cells (DCs). We used immature DCs differentiated from porcine blood monocytes under in vitro conditions. Based on the surface expression of MHC II and costimulatory CD80/86 molecules, we showed that both used EPSs favour the maturation of monocyte-derived DCs (MoDCs) similarly to the commonly used stimulant tumour necrosis factor α (TNF-α). In contrast to TNF-α stimulation, MoDCs treated with both used EPSs significantly up-regulated the mRNA levels not only for interleukin (IL)-10 (*P* < 0.0001 for EPS-DSM17938; *P* = 0.0037 for EPS-L26), but also for IL-12 (*P* = 0.0176 for EPS-DSM17938; *P* = 0.0019 for EPS-L26). These cytokines are known to regulate T-cell kinetics and play a key role in maintaining immune homeostasis. Interestingly, only relatively linear α-D-glucan (EPS-DSM17938) significantly increased gene expression of the major pro-inflammatory cytokine IL-1β (*P* = 0.0011) and the “SOS” cytokine IL-6 (*P* = 0.0127). However, it is important to highlight the need for further studies aimed at cytokine kinetics in DCs, as well as a co-culture study with allogenic T-lymphocytes.

**Supplementary Information:**

The online version contains supplementary material available at 10.1007/s11259-024-10445-6.

## Introduction

Immunomodulators of natural origin are being widely investigated for their use in food animals to improve productivity and animal health status, thereby minimizing antibiotic therapy and improving public health. Lactic acid bacteria-derived exopolysaccharides (LAB-EPSs) have recently gained widespread interest and popularity in this field of science.

LAB-EPSs are viscous, water-soluble, and high molecular weight carbohydrates that vary in saccharide composition and spatial conformation. Probiotic-derived EPSs as dietary supplements should meet certain criteria, such as undigestibility at the level of the stomach and small intestine, and they must be utilized by the resident gut microbiota in the colon (Oerlemans et al. 2021). Many studies have reported that LAB or their products have beneficial effects on the intestinal barrier function (Liu et al. 2020), and modulate the immune response of the intestinal mucosa (Cristofori et al. 2021). Mudroňová et al. (2018) assumed that among LAB products, EPSs are the most important immunomodulatory agents. In this work, EPSs originated from *L. reuteri* L26 Biocenol™ (EPS-L26) and *L. reuteri* DSM 17938 (EPS-DSM17938) were used to study as immunomodulators. Both are α-D-glucan polysaccharides with high molecular weight (820 kDa for EPS-L26; 650 kDa for EPS-DSM17938), but differing in degree of branching (Kšonžeková et al. 2016). The immunomodulatory effects of LAB-EPSs depend on their physiochemical properties, but mainly on their size and charge. While negatively charged EPSs and small molecules have a stimulatory effect, neutral and large EPSs act as suppressors. Of course, there are many contradictions in this research topic due to the use of different in vivo or in vitro models and different experimental design in each study (Werning et al. 2022). In general, LAB-EPSs have been reported to be able to activate different immune cells (macrophages, lymphocytes, DCs), modulate cytokine production also in non-immune cells, influence naive T-cell differentiation and maintain immune homeostasis (Saadat et al. 2019). DCs represents an unique class of immune cells which have a pivotal bridging role between innate and adaptive immunity. Activated/mature DCs migrate to the local lymph nodes or to the other lymphoid tissues, where they ensure the differentiation of naive T-lymphocytes into mature ones (Lebeer et al. 2008) according to their distribution, transcriptional programming and microenvironment (Zheng et al. 2021).

As mentioned above, bacterial EPSs contribute to the modulation of immune responses by influencing of the cytokine profile of many immune or non-immune cells (Saadat et al. 2019) but especially important is their interaction with professional antigen-presenting cells (APCs) (Pérez-Ramos et al. 2016; Kiššová et al. 2024). To study the EPS-induced immunomodulation of the professional APCs, we used porcine MoDCs, because of pigs are one of the major food animals and their immune system is the closest to that of humans. The immature MoDCs were differentiated from blood monocytes under in vitro conditions. This model represents an alternative approach to study the immunobiology of DCs, overcoming the low frequency of primary DCs and the difficulty of their isolation. The aim of the present study was to evaluate the cytokine response (mRNA level) of porcine MoDCs to both EPSs and their effect on the surface expression of selected MoDCs maturation markers.

## Materials and methods

### Bacterial strains and LAB-EPSs

*L. reuteri* L26 Biocenol™ (CCM 8616, isolated by MVDr. Radomíra Nemcová, PhD.; Department of Microbiology and Immunology, University of Veterinary Medicine and Pharmacy in Košice, Slovakia) and *L. reuteri* DSM17938 (BioGaia AB, Protectis, Sweden) were cultured according to Sims et al. (2011). EPSs for the present study were isolated and purified as described in an our previous report (Kšonžeková et al. 2016), where their structural characterization was published in detail. EPS-L26 (820 kDa) is a more branched α-D-glucan with (1→3) and (1→6) glycosidic linkages in ratio 1.3:1, while EPS-DSM17938 (650 kDa) is a relatively linear α-D-glucan with (1→4) and (1→6) glycosidic linkages in ratio 2.4:1.

### Porcine MoDCs generation

 Blood samples were collected from clinically healthy crossbred Landrace x Noble pigs from the supraorbital sinus, at the Clinic of Swine, at the University of Veterinary Medicine and Pharmacy in Košice. Briefly, peripheral blood mononuclear cells were isolated by density centrifugation on separation medium (LSM1077; PAA, Austria) as previously described (Kiššová et al. 2024). Monocytes were then isolated from the mononuclear fraction by positive magnetic sorting with anti-mouse IgG magnetic beads (New England BioLabs, England) and a mouse monoclonal antibody specific for the porcine CD14 (kindly provided from RNDr. Jiří Šinkora, PhD, Department of Immunology and Gnotobiology, Academy of Sciences of the Czech Republic, Prague). The purity of the isolated cells was checked by flow cytometry. The negligible contamination by lymphocytes (up to 10%) was observed in the monocyte suspension. For the generation of MoDCs, monocytes (100% CD14^+^, 71.8% MHC-II^+^,0% CD80/86^+^) were seeded as follows: 1 × 10^6^ cells/mL; 3 × 10^5^ cells/cm^2^. Subsequently, monocytes were cultured in RPMI medium (Sigma-Aldrich, USA) supplemented with 2 mM L-glutamine (Lonza, Switzerland), 10% FBS (heat-inactivated; Lonza, Switzerland), 1% penicillin/streptomycin (PAA, Austria) and porcine cytokines rpGM-CSF (20 ng/mL; R&D Systems, USA) and rpIL-4 (50 ng/mL; R&D Systems, USA) at 37 °C in a fully humidified 5% CO_2_ atmosphere for 6 days. The cell medium was changed every 2 days of cultivation.

### Experimental design

 To study the effect of LAB-EPS on the maturation of MoDCs, immature MoDCs were seeded at 2 × 10^5^ cells per well in 24-well culture plates (TPP, Switzerland). EPS were added to the MoDCs in a complete RPMI medium (2 mM L-glutamine, 10% FBS, 1% penicillin/streptomycin) at a final concentration 1 mg/mL. The cytokine TNF-α (5 ng/mL, R&D Systems, USA) was used as a positive control. Porcine immature MoDCs were stimulated with EPSs or TNF-α for 48 h. For gene expression analysis, cells were lysed and stored in a PureZole™ reagent (Bio-Rad, Hercules, CA). Surface expression of CD14, MHC II and costimulatory CD80/CD86 molecules was immediately evaluated using flow cytometry.

### Gene expression analysis

 Total RNA from MoDCs was isolated using a PureZole™ according to the manufacturer´s instructions and stored at -80 °C. RNA samples were treated with a DNA Rapid Removal Kit (Thermo Scientific, Waltham, MA, USA) and total RNA concentration and purity were determined using Nanodrop 8000 (Thermo Scientific, Waltham, MA). First-strand cDNA was constructed using RevertAid Premium Reverse Transcriptase with oligo(dT)18 and random hexamer primers (Thermo Scientific, Waltham, MA, USA). Primers are listed in Supplementary Table 1. Real-time PCR was performed in an iCycler CFX96 (Bio-Rad, Hercules, CA) in a 20 µl final volume containing 1 × iQ SYBR® Green Supermix (0.2 mM dNTP, 3 mM MgCl_2_), 0.5 µM of each primer and 35 ng of cDNA sample. The following protocol was used to study the expression of target genes by mRNA analysis: 95 °C for 5 min, 40 × [94 °C for 30 s, 60 °C (actin, IL-1β, IL-6, TGF-β, IL-12p35, IL-10) or 62 °C (actin; CD80) for 30 s, 72 °C for 30 s with fluorescence detection], 72 °C for 15 min. The final extension was followed by a melting curve analysis. Each assay included a calculation of reaction efficiency (100 ± 5%) and negative control without a cDNA template. Relative normalized expression was calculated by the 2^−ΔΔCt^ method using CFX96 Manager software (Bio-Rad, Hercules, CA) with actin as the reference gene (Kšonžeková et al. 2016).

### Flow cytometric analysis

 Flow cytometric analysis was used to evaluate the surface expression of CD14, MHC II and CD80/CD86 costimulatory molecules. The primary and secondary antibodies are listed in Supplementary Table 2. Cells were incubated with primary antibodies for 20 min at room temperature. The cells were then washed and incubated with secondary antibodies (1:100 dilution; BIO-PORT Europe, Czech Republic) for 15 min at room temperature. Finally, cells were washed twice with PBS and analyzed on a BD FACS Canto flow cytometer (BD Biosciences, USA) using BD FACS Diva software (Kiššová et al. 2024). Cell position was defined in FSC-A vs. SSC-A dot plot. Doublets and cell aggregates were removed from the analysis using the SSC-A vs. SSC-H dot plot. The percentage of MHCII + cells and their mean fluorescence intensity (MFI) for APC were evaluated in the histogram (APC-A vs. counts). Determination of the percentage of CD80/86 + and MHCII + cells was performed in the PE-A vs. APC-A dot plot (Fig. 2).

### Statistical analysis

 Results are expressed as mean *±* standard deviation (SD, *n* = 3). Significant differences were determined by one-way analysis of variance (ANOVA) followed by Dunnett’s multiple comparison test using GraphPad Prism 9.0.0 software.

## Results and discussion

In our work a heterogeneous culture of porcine immature MoDCs consisting of adherent and non-adherent cell subpopulations was used. The non-adherent or weakly adherent subpopulation was dominated by cells with dendritic-like veiled processes, but the adherent subpopulation also had a macrophage-like morphology (Supplementary Fig. 1). In general, porcine MoDCs are phenotypically characterized as CD1^+^, CD14^+^, CD16^+^, CD80/86^+^, CD172^+^ and MHC II^+^ cells. In our experiment, the MoDCs had characteristics of an immature phenotype, with an average surface expression of MHC II molecules (MFI: 33 800) and low levels of the costimulatory molecules CD80/86 (MFI: 8 200). During DC maturation, the expression of these cell surface markers, which play a key role in triggering the T-cell immune responses, increases significantly (Carrasco et al. 2001; Zheng 2021). We found a similar stimulation of these maturation markers (MFI for MHC II^+^: 86 056; MFI for CD80/86^+^: 19 300) with the continued presence of CD14 after TNF-α treatment of porcine MoDCs as shown in supplementary Fig. 1D. However, partial activation was observed even when the MoDCs were not stimulated (Fig. 2; negative control: MFI for MHC II^+^: 68 755; MFI for CD80/86^+^: 15 750), due to mechanical manipulation and continued culturing.

**Fig. 1:**
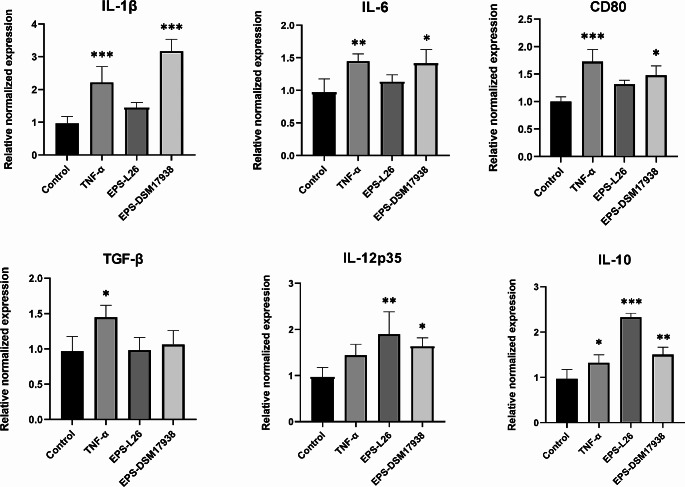
The effect of EPS produced by *Limosilactobacillus reuteri* L26 Biocenol™ (EPS-L26) and *L. reuteri* DSM 17938 (EPS-DSM 17938) on transcriptional answer of selected genes (interleukine (IL)-1β, IL-6, IL-10, IL-12, TGF-β and CD80) in porcine dendritic cells (DCs). Immature monocyte-derived DCs (MoDCs) were stimulated with these EPSs for 48 h. Results are expressed as mean ± standard deviation (*n* = 3). Statistical significance was assesed by Dunnet’s multiple comparison test. Significantly different from control (*p < 0.05; **p < 0.01; ***p < 0.001)

In the present study, immature/non-activated MoDCs were treated with EPSs isolated from two different *L. reuteri* strains (EPS-L26, EPS-DSM17938). The cytokine TNF-α was used as a positive control. TNF-α treatment of immature MoDCs induced the gene expression of CD80 and the cytokines IL-1β, IL-6, TGF-β and IL-10 (Fig. 1). Regarding the immunoregulatory potential of the EPSs tested, we found that both significantly up-regulated IL-12p35 and IL-10 mRNA (Fig. 1). In the study of Tang et al. (2015), EPS derived from *Lactiplantibacillus plantarum* also stimulated immature DCs (from bone marrow; BMDCs) and increased IL-12p70 secretion in a dose-dependent manner. However, they showed an inhibitory effect of this EPS on IL-10 production, which is in contrast to our results. Similar to our results, EPS of *Lacticaseibacillus rhamnosus *KL37 stimulate IL-10 production in murine peritoneal macrophages in a dose-dependent manner (Ciszek-Lenda et al. 2011). Christensen et al. (2002) investigated the effect of six LAB strains on the maturation of BMDCs and the most effective were those with the greatest ability to induce IL-12 production. In general, activated APCs are the main producers of IL-10 and IL-12, which play a key role in maintaining immune homeostasis. As one of them is an anti-inflammatory and the other is pro-inflammatory, they possess opposite functions, but the ratio of these two cytokine levels regulates T-cell kinetics, which can be affected during immune responses (Ma et al. 2015).

Both polysaccharides tested in this study are high molecular weight α-D-glucans with differences in spatial conformation. Interestingly, the relatively linear EPS-DSM17938 increased gene expression of the major pro-inflammatory cytokine IL-1β and the ‘SOS’ cytokine IL-6 (Fig. 1) and this is the only difference in the cytokine profile between these EPSs. *Lactobacillus helveticus* produces heteropolysaccharides with similar effects in other professional APCs. Specifically, it stimulates the production of IL-6, IL-1β and TNF-α in the murine macrophage cell line RAW264.7 (You et al. 2020). In our recently published study (Kiššová et al. 2024), the short-term treatment (4 h) of porcine MoDCs with EPS-L26 also resulted in a stimulation of the expression of the IL-6 gene in porcine MoDCs. However, this was only observed in monoculture and not in co-culture model with IPEC-J2 epithelial cells.

**Fig. 2:**
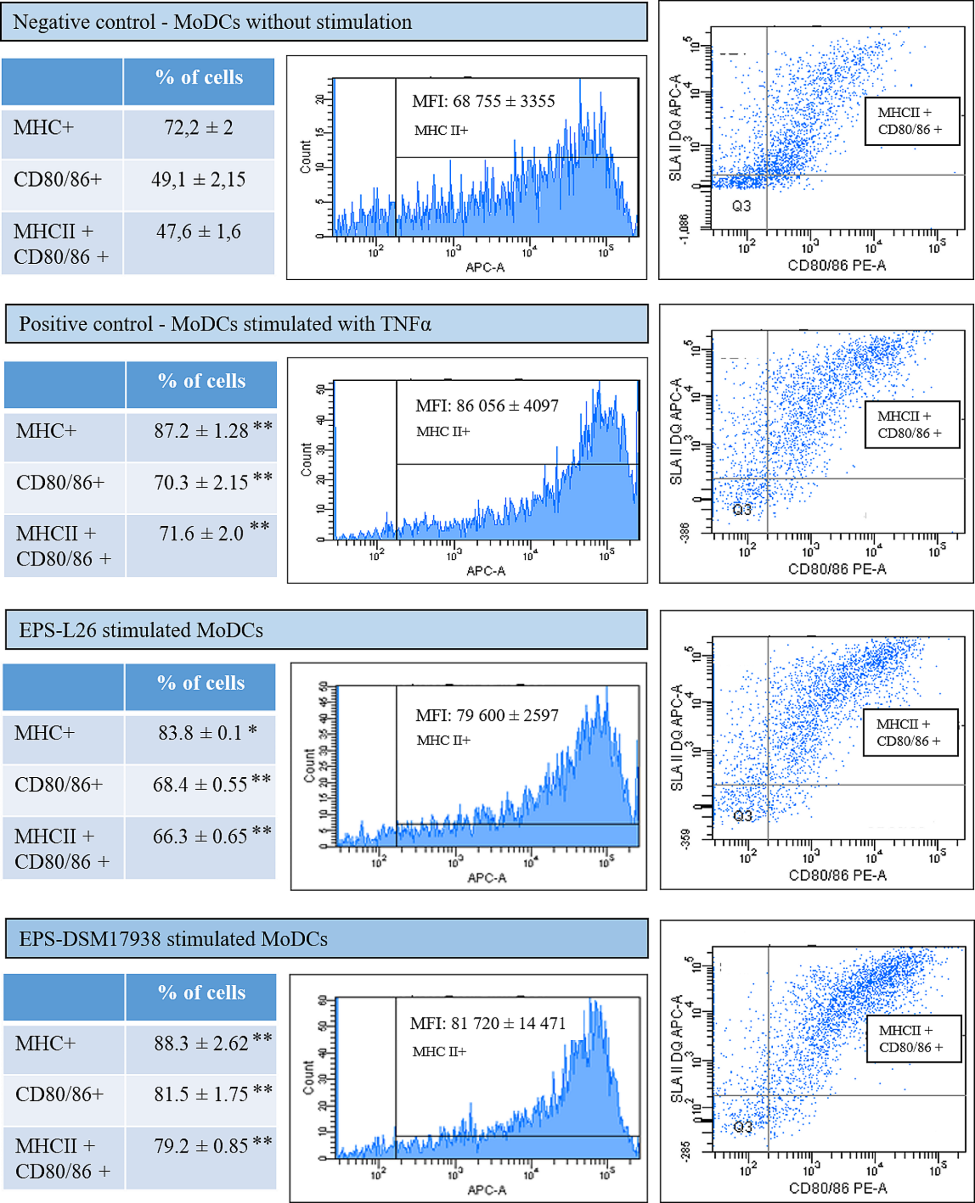
EPS produced by *Limosilactobacillus reuteri* L26 Biocenol™ (EPS-L26) and *L. reuteri* DSM 17938 (EPS-DSM 17938) stimulate the surface expression of dendritic cell (DC) maturation markers (MHC II, CD80/86). Immature monocyte-derived DCs (MoDCs) were treated with these EPS (1 mg/mL) for 48 h. Results are expressed as mean ± standard deviation (*n* = 3). Statistical significance was assesed by Dunnet’s multiple comparison test. Significantly different from negative control (*p < 0.05; **p < 0.01). MFI - mean fluorescence intensity; TNFα - tumour necrosis factor α; APC - allophycocyanin; PE - phycoerytrin; SLA-II - MHC II

To determine the influence of EPS-L26 and EPS-DSM17938 on the immunophenotype of porcine MoDCs, the selected surface markers (CD14, MHC II, CD80/86) were evaluated using flow cytometry. Both EPSs significantly increased the surface expression of the DC maturation markers (MHC II, CD80/86) to a similar extent as the cytokine TNF-α (positive control; Fig. 2). Based on our results, it appears that the structure of the α-D-glucans in terms of the degree of branching does not have an effect on their ability to mature MoDCs. To the best of our knowledge, only a few studies have investigated the effect of LAB-EPS on the DCs response. EPS produced by *Lactiplantibacillus plantarum* (Tang et al. 2015) or *Lacticaseibacillus casei* (Ren et al. 2019) similarly up-regulated the surface expression of MHC II and CD86 on mouse BMDCs, and thus activated BMDCs promoted the proliferation of allogenic T-cells in vitro (Tang et al. 2015). The EPS-induced activation of APCs with subsequent production of cytokines, chemokines and up-regulation of costimulatory molecules can be mediated by Toll-like receptors (TLR-2,TLR-4) or C-type lectin receptor (DC-SIGN) in DCs (Saadat et al. 2019).

Recently, LAB have been widely tested for their effect on DC maturation using MoDCs (Mohamadzadeh et al. 2005; Zheng et al. 2021) or BMDCs (Christensen et al. 2002; Foligne et al. 2007) as an in vitro experimental model. *Lactobacillus acidophilus*, *L. lactis*, *Lacticaseibacillus casei*, *Lactobacillus gasseri*, *Limosilactobacillus reuteri* and *Lactobacillus johnsonii* were able to activate DCs as shown by up-regulation of MHC II., CD40, CD80, CD83, CD86 or CD172 (Christensen et al. 2002; Mohamadzadeh et al. 2005; Foligne et al. 2007; Zheng et al. 2021). Interestingly, the surface expression of co-stimulatory markers (CD40, CD80) was low in BMDCs treated with *Ligilactobacillus salivarius* or *Lacticaseibacillus rhamnosus*. It appears that different *Lactobacillus* species or their products have very different effects on DC maturation and further research is needed in this area of science.

In conclusion, through analyzing the expression of surface markers such as MHC II and costimulatory CD80/86 molecules, we demonstrated that the both tested α-D-glucans (EPS-L26, EPS-DSM17938) derived from different *L. reuteri* strains can effectively induce the maturation of MoDCs in a manner similar to the commonly used stimulant TNF-α. Interestingly, both tested EPS significantly increased mRNA levels of the antagonistic cytokines IL-10 and IL-12, which play a key role in maintaining immune homeostasis. The relatively linear α-D-glucan (EPS-DSM17938) also significantly increased gene expression of the major pro-inflammatory cytokine IL-1β (*P* = 0.0011) and the “SOS” cytokine IL-6 (*P* = 0.0127). The EPSs produced by *L. reuteri* exhibit immunomodulatory properties, suggesting their potential utility in various commercial applications such as dietary supplements, prophylactics, or vaccine adjuvants.

## Electronic supplementary material

Below is the link to the electronic supplementary material.

Supplementary Material 1 (DOCX 14.6 KB)

Supplementary Material 2 (DOCX 13.3 KB)

Supplementary Material 3 (PNG 773 KB)

High Resolution Image (TIF 1.16 MB)

## Data Availability

No datasets were generated or analysed during the current study.
