# Supplementary material for: Exopolysaccharides from Limosilactobacillus reuteri: their influence on in vitro activation of porcine monocyte-derived dendritic cells - brief report
Source: Vet Res Commun. 2024 Jul 4;48(5):3315–21. doi: 10.1007/s11259-024-10445-6 (PMC11442659; doi:10.1007/s11259-024-10445-6)
Supplement: Supplementary file 1 — Supplementary Material 1 (DOCX 14.6 KB) [file 11259_2024_10445_MOESM1_ESM.docx]

***Supplementary table 1: The primers used in the study.***

|  | **Forward primer (5´-3´)** | **Reverse primer (5´-3´)** | **Acc**. **number** | **Product lenght** | **Reference** |
| --- | --- | --- | --- | --- | --- |
| **β-actin** | CAT CAC CAT CGG CAA CGA | GCG TAG AGG TCC TTC CTG ATG T | U07786 | 143 bp | Moue et al. (2008) |
| **IL-1β** | GCC CTG TAC CCC AAC TGG TA | CCA GGA AGA CGG GCT TTT G | M86725 | 61 bp | Kšonžeková et al. (2016) |
| **IL-6** | TGG ATA AGC TGC AGT CAC AG | ATT ATC CGA ATG GCC CTC AG | M86722 | 109 bp | Moue et al. (2008) |
| **CD80** | CCC CCA GGG GCA GTT GGT GT | CAC TGA TGG GTG GGG CCA GA | NM 214087.1 | 300 bp | This study |
| **TGF-β** | CAC GTG GAG CTA TACCAG AA | TCC GGT GAC ATC AAA GGA CA | Y00111 | 108 bp | Moue et al. (2008) |
| **IL-12p35** | AGT TCC AGG CCA TGA ATG CA | TGG CAC AGT CTC ACT GTT GA | L35765 | 125 bp | Moue et al. (2008) |
| **IL-10** | CCG GGA ACT CCG AGC TGC CT | ATT GAT GAC AGC GCC GCA GC | NM214041 | 263 bp | This study |

References:

Kšonžeková P, Bystrický P, Vlčková S, Pätoprstý V, Pulzová L, Mudroňová D et al (2016) Exopolysaccharides of Lactobacillus reuteri: Their influence on adherence of E. coli to epithelial cells and inflammatory response. Carbohydr Polym 141:10–19. doi: 10.1016/j.carbpol.2015.12.037

Moue M, Tohno M, Shimazu T, Kido T, Aso H, Saito T, Kitazawa H (2008) Toll-like receptor 4 and cytokine expression involved in functional immune response in an originally established porcine intestinal epitheliocyte cell line. Biochim Biophys Acta 1780(2):134-44. doi: 10.1016/j.bbagen.2007.11.006
