# Supplementary material for: Exopolysaccharides from Limosilactobacillus reuteri: their influence on in vitro activation of porcine monocyte-derived dendritic cells - brief report
Source: Vet Res Commun. 2024 Jul 4;48(5):3315–21. doi: 10.1007/s11259-024-10445-6 (PMC11442659; doi:10.1007/s11259-024-10445-6)
Supplement: Supplementary file 2 — Supplementary Material 2 (DOCX 13.3 KB) [file 11259_2024_10445_MOESM2_ESM.docx]

***Supplementary table 2: Primary and secondary antibodies used in the study.***

| **Primary antibody** | **Clone** | **Dilution** | **Secondary antibody** | **Source** |
| --- | --- | --- | --- | --- |
| ***Anti-Porcine CD14*** | MIL-2 | 1:200 | *Anti-Mouse IgG2b FITC* | Dr. Šinkora |
| ***Anti-Porcine MHC Class II DQ*** | K274.3G8 | 1:30 | *Anti-mouse IgG1 APC* | AbD Serotec, USA |
| ***Human CD152(CTLA-4) Ig/Fusion Protein*** | - | 1:500 | *Anti-mouse IgG2a PE* | Ancell, USA |

*FITC – fluorescein-isothiocyanate, APC - allophycocyanin, PE - phycoerythrin*
