# Supplementary figures and images for: Exopolysaccharides from Limosilactobacillus reuteri: their influence on in vitro activation of porcine monocyte-derived dendritic cells - brief report
Source: Vet Res Commun. 2024 Jul 4;48(5):3315–21. doi: 10.1007/s11259-024-10445-6 (PMC11442659; doi:10.1007/s11259-024-10445-6)

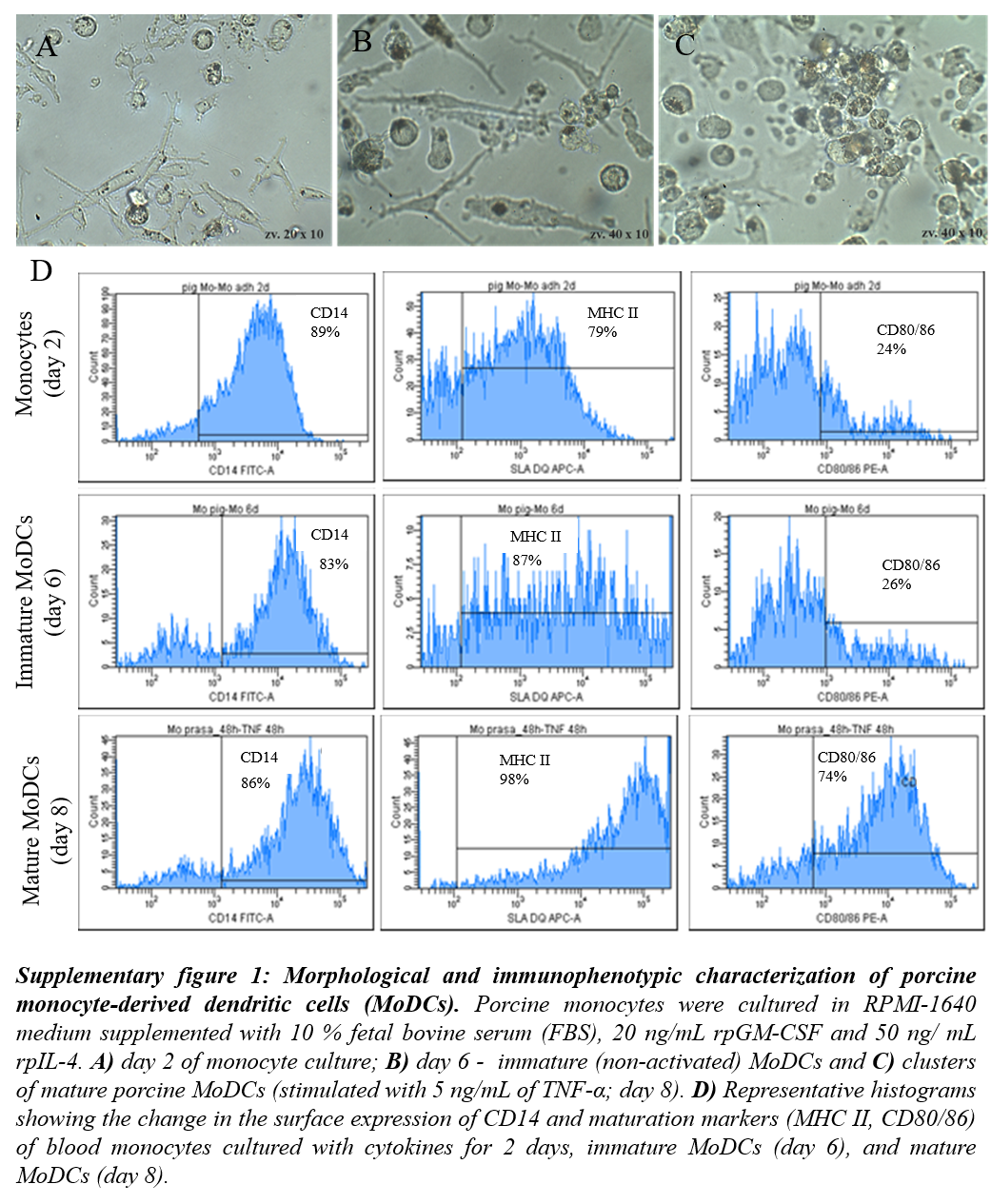

Supplement: Supplementary file 3 — Supplementary Material 3 (PNG 773 KB) [file 11259_2024_10445_Fig3_ESM.png]

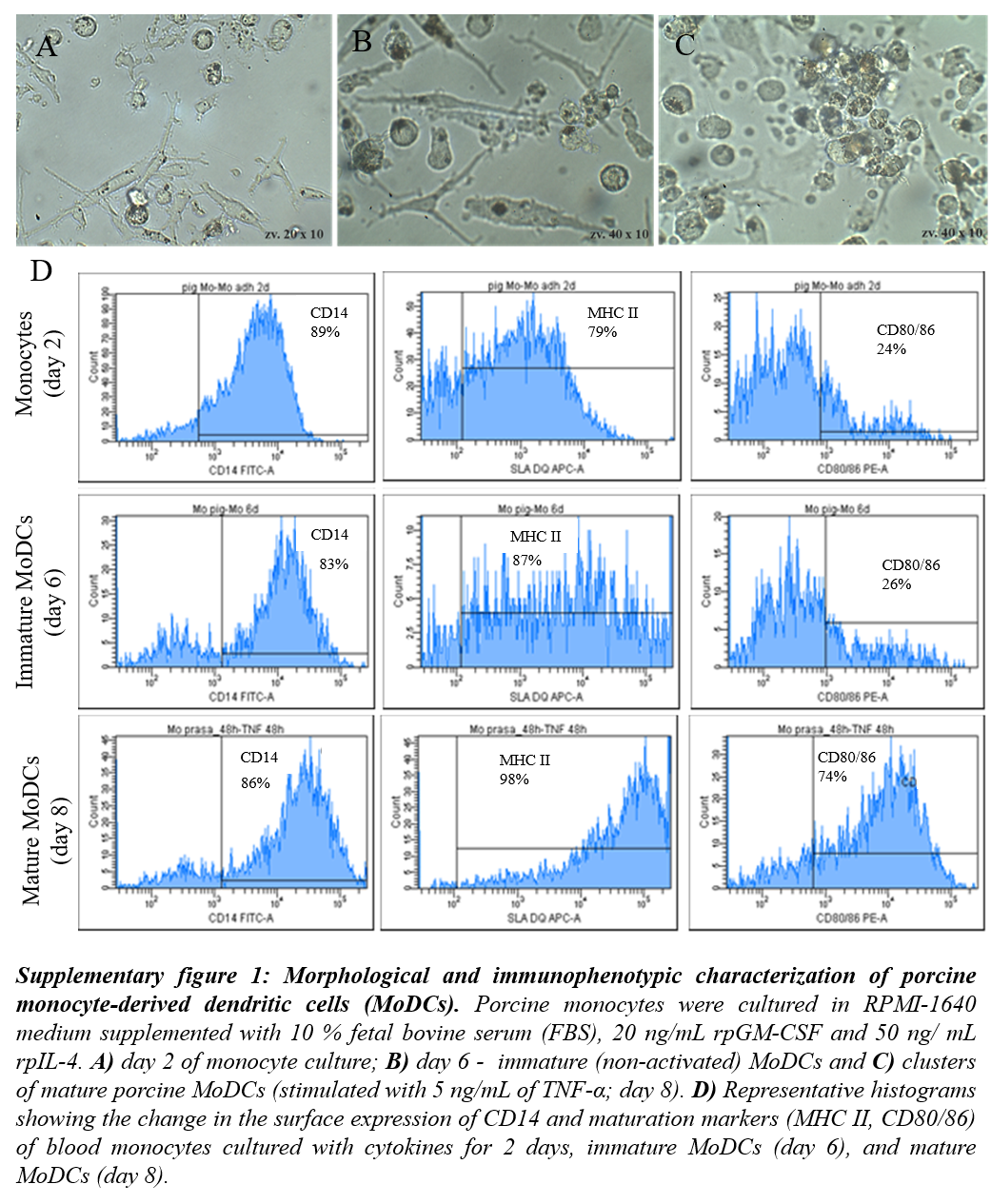

Supplement: Supplementary file 4 — High Resolution Image (TIF 1.16 MB) [file 11259_2024_10445_MOESM4_ESM.tif]
